# Effects of Precipitation Increase on Soil Respiration: A Three-Year Field Experiment in Subtropical Forests in China

**DOI:** 10.1371/journal.pone.0041493

**Published:** 2012-07-23

**Authors:** Qi Deng, Dafeng Hui, Deqiang Zhang, Guoyi Zhou, Juxiu Liu, Shizhong Liu, Guowei Chu, Jiong Li

**Affiliations:** 1 South China Botanical Garden, Chinese Academy of Sciences, Guangzhou, China; 2 Wuhan Botanical Garden, Chinese Academy of Sciences, Wuhan, China; 3 Department of Biological Sciences, Tennessee State University, Nashville, Tennessee, United States of America; DOE Pacific Northwest National Laboratory, United States of America

## Abstract

**Background:**

The aim of this study was to determine response patterns and mechanisms of soil respiration to precipitation increases in subtropical regions.

**Methodology/Principal Findings:**

Field plots in three typical forests [i.e. pine forest (PF), broadleaf forest (BF), and pine and broadleaf mixed forest (MF)] in subtropical China were exposed under either Double Precipitation (DP) treatment or Ambient Precipitation (AP). Soil respiration, soil temperature, soil moisture, soil microbial biomass and fine root biomass were measured over three years. We tested whether precipitation treatments influenced the relationship of soil respiration rate (*R*) with soil temperature (*T*) and soil moisture (*M*) using *R = *(*a+cM*)exp(*bT*), where *a* is a parameter related to basal soil respiration; *b* and *c* are parameters related to the soil temperature and moisture sensitivities of soil respiration, respectively. We found that the DP treatment only slightly increased mean annual soil respiration in the PF (15.4%) and did not significantly change soil respiration in the MF and the BF. In the BF, the increase in soil respiration was related to the enhancements of both soil fine root biomass and microbial biomass. The DP treatment did not change model parameters, but increased soil moisture, resulting in a slight increase in soil respiration. In the MF and the BF, the DP treatment decreased soil temperature sensitivity *b* but increased basal soil respiration *a*, resulting in no significant change in soil respiration.

**Conclusion/Significance:**

Our results indicate that precipitation increasing in subtropical regions in China may have limited effects on soil respiration.

## Introduction

Soil respiration in terrestrial ecosystems plays an important role in global carbon cycling and climate change [1]–[4]. However, our understanding of precipitation impacts on soil respiration is still very limited, particularly in tropical and subtropical forests [5]. As greater intensity of precipitation and more severe droughts and floods are predicted in the future [6]–[7], such changes in precipitation may have significant influences on soil moisture and soil respiration in terrestrial ecosystems. Compared to drought, few studies have been done on the influence of heavy precipitation on soil respiration [8]–[14]. Considering that tropical and subtropical forests contain more than 25% of the carbon in the terrestrial biosphere, it is imperative to improve our mechanistic understanding of soil respiration responses to precipitation and soil moisture changes [15], [16].

Soil respiration includes both respiration of living roots and microbial respiration resulted from microbial decomposition of litter and soil organic matter [3], [5], [10], [12]. Root activity and microbial decomposition are often subject to both environmental factors and substrate changes related to phenological processes [17]–[20]. Any changes in root biomass, soil organic matter, root and microbial activities due to precipitation change could influence soil respiration. Like many biological processes, soil respiration is also influenced by soil temperature and moisture in many different ecosystems [3], [21]–[23]. While it is generally accepted that global warming could influence the relationship of soil respiration and temperature, how precipitation treatments would influence soil respiration and its relationship to soil moisture has not been well investigated. When treatments such as warming, precipitation, or CO_2_ concentration changes are applied, response variables may respond directly to changes in environmental factors as well as alter their relationships with environmental factors. Thus, soil respiration responses to precipitation treatments could be caused by either changes in environmental factors such as soil temperature and moisture, or functional changes – which are defined as changes in model parameters of soil respiration with soil temperature and moisture, or both [23]. For example, functional change due to a change in soil temperature sensitivity may increase or decrease soil respiration even when soil temperature is not influenced by precipitation treatments. Functional change could be attributed to the changes in phenological process, substrate or microbial activity in an ecosystem [2], [5], [16], [23].

Changes in soil moisture under different precipitation treatments could influence the responses of soil respiration to precipitation. There is no doubt that precipitation is usually the driving factor of the dynamics in soil moisture. However, soil water storage after precipitation events depends on vegetation types and covers, soil characteristics (e.g., infiltration rates, slopes, textures, depths, impermeable layers), and losses to deep drainage, lateral flow, and evaporation [24]. Thus, the response of soil moisture to precipitation treatments often varies in different ecosystems. For example, drought treatments using automated retractable curtains reduced soil moisture by 32–48%, 15–61%, and 19–25% at three heathlands [25], and double precipitation increased soil moisture by only 10% in Oklahoma grassland [10]. How precipitation changes influence soil moisture in subtropical forests may have significant impacts on soil respiration.

Functional changes (i.e. changes in model parameters of soil respiration with soil temperature and moisture) reflect underlying biological changes in the response of soil respiration to precipitation changes. Many empirical models of soil respiration and soil moisture have been developed [26]–[29]. Response of soil respiration to soil moisture is usually nonlinear, with soil respiration increases with soil moisture increases, levels off at high soil moisture, and even decreases when soil moisture is too high [3], [28], [29]. However, linear regression seems to work well in many different ecosystems, including boreal forests, sub-Antarctic island ecosystems, temperate grasslands, temperate forests, Mediterranean ecosystems, and particularly, tropical and subtropical forests [22], [30]–[34]. The slope of the linear regression model can be considered as soil moisture sensitivity, as it reflects an average change in soil respiration due to one unit change of soil moisture. While many precipitation manipulation experiments have been performed [9]–[15], [21], [22], only a few studies have attempted to study the soil moisture sensitivity change under climate change, particularly precipitation [3], [35]–[37].

Another important functional relationship is the response of soil respiration to soil temperature [2], [38], [39]. Soil temperature is the major control of soil respiration due to its influences on the kinetics of microbial decomposition, root respiration and diffusion of enzymes and substrates [32], [40]. Numerous studies have focused on the responses of soil respiration to soil temperature. The most widely used model is an exponential equation (*R = R_0_*exp(*bT*)) where *R* is soil respiration, *T* is soil temperature, and parameter *R_0_* is basal soil respiration, and *b* is related to soil temperature sensitivity (*Q_10_* = exp(10*b*) [41], [42]. Many studies reported that soil temperature sensitivity may decrease under high temperature treatments [2], [30], [38], [39] and increase under low temperature [38], [40]–[43]. Several studies also indicated that soil water stress or excess may decrease soil temperature sensitivity of soil respiration [27], [42], [44]. Since soil temperature and soil moisture may interactively regulate soil respiration in field conditions, relationships of soil respiration with both soil temperature and moisture have also been proposed [20], [36], [45]. Whether and how soil moisture and temperature sensitivities vary with precipitation increase have not been well investigated [5], [14].

We conducted a precipitation manipulation field experiment in subtropical forests in Southern China with an overall aim to understand the responses of soil respiration to precipitation increase. We selected three common forests at the study site, established two precipitation treatments in each forest, and measured soil respiration over three years. Double precipitation was realized through automatic interception-redistribution systems that delivering intercepted precipitation from nearby plots of the same size [10]. Adjacent control plots received ambient precipitation (AP). We addressed the following three questions in this study: 1) what are the response patterns of soil respiration to precipitation increase in the subtropical forests? 2) Do different forest sites respond differently to precipitation increase? 3) Does precipitation increase influence soil temperature and moisture sensitivities? The conclusions obtained in this study will enrich our knowledge of soil respiration responses to precipitation changes in subtropical forests in China and may have potentially significant implications for terrestrial ecosystem carbon cycling.

## Materials and Methods

### Ethics Statement

The study site is maintained by the South China Botanical Garden, Chinese Academy of Sciences. The location is within the Dinghushan Forest Ecosystem Research Station, Chinese Ecosystem Research Network (CERN). All necessary permits were obtained for the described field study. The field study did not involve endangered or protected species. Data will be made available upon request.

### Site Description

The study site is located in the center of Guangdong Province in southern China (112°13′39′′–112°33′41′′ E, 23°09′21′′–23°11′30′′ N). Climate in the region is typical south subtropical monsoon climate, with mean annual temperature of 21.4°C, and mean annual precipitation of 1956 mm [33], of which nearly 80% falls in the hot-humid wet/rainy season (April-September) and 20% in the dry season (October-March). The bedrock is sandstone and shale. Three common subtropical forests (at elevations ranging from 200 to 300 m, less than 500 m from one another and facing the same slope direction) were selected including a coniferous Masson pine forest (PF), a conifer and broadleaf mixed forest (MF), and an evergreen broadleaf forest (BF). The three forests also represent forests in early-, middle-, and advanced-successional stages in the region [46], [47]. Soil properties and major stand information are listed in Table 1. The PF (approximately 22 ha), originally planted by local people in the 1950 s, was dominated by *Pinus massoniana* in the tree layer and *Baeckea frutescens*, *Rhodomyrtus tomenosa*, and *Dicranopteris linearis* in the shrub and herb layers. The MF (approximately 557 ha) was developed from artificial pine forest with a gradual invasion of some pioneer broadleaf species through natural succession. The upper canopy of the community is dominated by *Schima superba*, *Castanopsis chinensis*, and *Craibiodendron scleranthum var. kwangtungense*. Artificial disturbances have not occurred in the MF for about 100 years. The BF (approximately 218 ha) located in the central area of the reserve was dominated by *Castanopsis chinensis*, *Cryptocarya concinna*, *Schima superba*, *Machilus chinensis* without any *Pinus massoniana*. No disturbance was recorded for the past 400 years in the BF [37]–[38].

**Table 1:** Stand characteristics of the pine forest (PF), the mixed forest (MF) and the broadleaf forest (BF) at the Dinghushan Forest Ecosystem Research Station.

Forests	PF	MF	BF
Elevation (m)	200–300	220–300	220–300
Stand age (year)	50–60	About 110	About 400
Successional stage	Early	Middle	Advanced
Biomass (Mg C ha^–1^)^a^	61.3	82.1	145.2
Standing litter (g m^–2^)^b^	436±146	497±103	328±71
Abovegroud litter input (g m^–2 ^yr^–1^)^b^	699±76	801±142	631±105
LAI^c^	4.3±0.4	6.5±0.7	7.8±0.5
SOM (0–10 cm) (g kg^–1^ soil)^c^	23.3±1.1	26.8±1.3	38.9±1.6
Bulk density (0–10 cm) (g cm^–3^)^c^	1.32±0.04	1.10±0.08	0.86±0.06
SOC (0–60 cm) (Mg C ha^–1^)^d^	105.2	111.3	164.1
Gravel (%)^e^	34.7	19.8	12.7
Sand (%)^e^	48.8	48.0	38.1
Silt (%)^e^	26.3	22.1	26.7
Clay (%)^e^	23.9	29.9	35.2
Soil pH value^f^	3.79±0.05	3.86±0.03	3.92±0.03

a From Liu *et al.* (2007) [57].

b Unpublished data from the Dinghushan Forest Ecosystem Research Station (2007–2009).

c From Zhang *et al.* (2006) [33]. SOM and LAI represent soil organic matter in the top 10 cm depth and leaf area index, respectively.

d From Fang *et al.* (2003) [60]. SOC represents soil organic carbon in the top 60 cm depth.

e From He and others (1982) [61].

f From Yan *et al.* (2009) [56].

### Experimental Design

We used a two-factor experimental design considering forest ecosystem type and precipitation treatment. At each forest site, a randomized block design was used with three blocks. In each block, one double precipitation (DP) treatment plot and one control plot were arranged. For the DP plot, precipitation was intercepted in a nearby plot with same size as the treatment plot using transparent polyvinyl chloride (PVC) sheer roof and was redistributed to the DP plot using pipes similar to those used in [10]. The control plot that received ambient precipitation (AP) was built next to the treatment plot. Each plot was 3×3 m^2^ and the distance between the DP and AP plots was more than one meter.

### Soil Respiration Measurements

Five PVC soil collars (80 cm^2^ in area and 5 cm in height) were permanently installed 3 cm into the soil in each plot in November 2006. The distance between adjacent collars was more than 50 cm. Soil respiration was measured three times a month from January 2007 to December 2008 and two times a month in 2009 using a Li-6400 infrared gas analyzer (Li-COR, Inc., Lincoln, Nebraska, USA) connected to a Li-6400-09 soil respiration chamber (9.55 cm diameter) (Li-COR, Inc., Lincoln, Nebraska, USA). The measurements were made between 9∶00 am and 12∶00 pm local time. Previous work at this study site has demonstrated that soil respiration in forests measured during this period was close to daily mean [30], [48]. Soil respiration was measured three cycles for each soil collar and the CO_2_ concentration change in the chamber to complete one cycle was set as 10 ppm above the set point. Soil respiration in a treatment plot was calculated as the mean of five collar measurements (the measurement at five collars in a plot mostly differed by less than 5% at any measurement period). Soil temperature at 5 cm below the soil surface was also monitored with a thermocouple sensor attached to the respiration chamber during the soil respiration measurement. Volumetric soil moisture of the top 5 cm soil layer was measured on five random locations within a treatment plot using a PMKit [34] at the same time when the soil respiration measurements were being taken.

### Soil Microbial Biomass and Fine Root Biomass Measurements

Soil samples were collected in February 2008 to determine soil microbial biomass C content, and three more times in May 2008, August 2008 and November 2008. Two samples of six cores (2.5 cm diameter) were randomly collected from each plot in the three forests. After removing roots and plant residues, the composited samples were immediately sieved through a 2-mm mesh sieve. The soil microbial biomass carbon was determined by the fumigation-extraction technique. The soil microbial biomass carbon was extracted with potassium sulfate on both fumigated and unfumigated soil [49], [50]. The carbon content of the extract was tested and the biomass was calculated based on the difference between the carbon content of fumigated vs. the unfumigated soil [49], [50].

To measure fine root biomass (diameter≤3 mm), we collected soil cores (0–20 cm depth) in February 2008 using a 10 cm diameter stainless-steel corer, and three more times in April 2008, August 2008 and October 2008. Each sample was randomly collected from each plot in each forest. Fine roots were separated by washing and sieving, dried at 60°C for 48 h and weighed.

### Statistical Analysis

Soil respiration rate and soil temperature in a plot were calculated as the means of five collar measurements. Soil moisture was calculated as the mean of five measurements at random locations in a plot. We used repeated measure Analysis of Variance (ANOVA) to test the differences in soil respiration rate, soil temperature and soil moisture among forests, precipitation treatments, and years. Each treatment was replicated three times (three blocks). Multiple comparisons (Least Significant Difference, LSD method) were conducted if significant effects of forest ecosystem types, precipitation treatments or years were found. Previous work at study sites demonstrated that soil respiration increases exponentially with soil temperature and linearly with soil moisture [30], [33], [34]. Thus, we first developed the relationship between soil respiration and soil temperature with an exponential function and the relationship between soil respiration and soil moisture with a linear regression mode. Considering that soil temperature and moisture may interactively regulate soil respiration, we also fit soil respiration (*R*) with soil temperature (*T*) and soil moisture (*M*) together using *R = *(*a+cM*)exp(*bT*), where *a* is parameter related to basal soil respiration when both *T* = 0 and *M* = 0; *b* and *c* are parameters related to the soil temperature and moisture sensitivities of soil respiration, respectively. Like most studies, we used measurements of soil respiration, soil temperature and moisture of whole years here. One caveat of this approach was that seasonal variations of tree roots growth, carbon substrate in the soils, and soil microbial community would influence soil respiration, but were difficult to quantify. Non-linear least square method was used to derive the model parameters using SAS NLIN procedure [51]. Soil temperature and moisture sensitivities were derived for different precipitation treatments in the three forests. All data analyses were carried out using SAS software Version 9.1 [51] (SAS Institute Inc., Cary, NC, USA).

## Results

### Effects of Precipitation Treatments on Soil Temperature and Moisture

**Figure 1:**
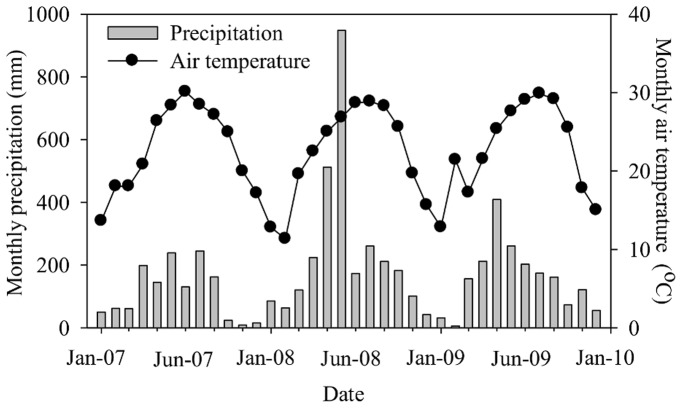
Monthly rainfall and mean air temperature at the Dinghushan Forest Ecosystem Research Station in Southern China during the experimental period from 2007 to 2009.

There were strong seasonal variations of precipitation in all three years, with intensive precipitation occurring from April through September (i.e., wet season) (Figure 1). The annual precipitation amount was 1341.6, 2925.8, and 1864.4 mm in 2007, 2008, and 2009, respectively. The very high precipitation in 2008 was mostly attributed to two heavy precipitation months (May and June) which had 50% of the total annual precipitation (Figure 1). The high precipitation intensity and large interannual variability in precipitation throughout the three years were typical in subtropical China. Mean annual air temperature did not vary much and was 22.77, 22.08, 22.71°C in 2007, 2008, and 2009, respectively. The monthly mean air temperature ranged from 11.35°C (February 2008) to 30.11°C (July 2007).

**Figure 2:**
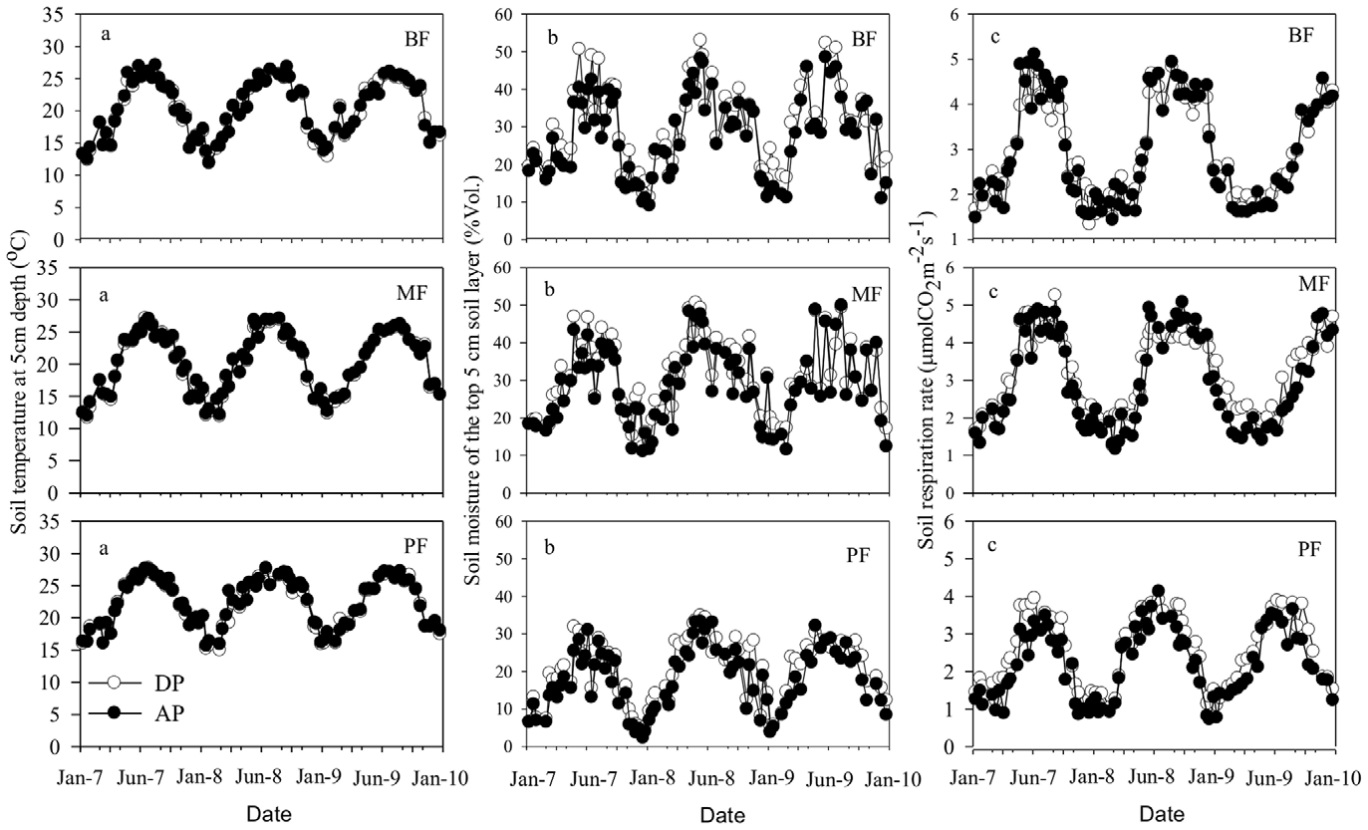
Seasonal dynamics of soil temperature at 5 cm depth, soil moisture of the top 5 cm soil layer, and soil respiration under ambient precipitation (AP) and double precipitation (DP) treatments in the broadleaf forest (BF), the mixed forest (MF) and pine forest (BF).

The seasonal patterns of soil temperature in three forests were similar to the pattern of air temperature (Figure 2a). Among the three forests, soil in the PF was significantly warmer (22.42°C) than that in the MF (20.20°C) and the BF (20.32°C) (Tables 2 and 3). No significant difference in annual mean soil temperature was found between the MF and the BF. Precipitation treatments did not change soil temperature in all three forests.

**Table 2:** Significance test using Analysis of Variance (ANOVA).

Source	df	Soil respiration	Soil temperature	Soil moisture
Forest	2	79.97^**^	41.88^**^	158.98^**^
Precipitation	1	11.56^**^	0.57	30.58^**^
Forest×Precipitation	2	2.97^*^	0.01	0.08
Year	2	0.18	0.39	25.24^**^
Forest×Year	4	1.45	0.10	0.75
Precipitation×Year	2	0.10	0.03	0.05
Forest×Precipitation×Year	4	0.08	0.02	0.25

Significance of the effects of forest type, precipitation treatment, year and their interactions on soil respiration rate, soil temperature, and soil moisture at the Dinghushan Forest Ecosystem Research Station are tested using ANOVA. Numbers are F-values. Stars indicate the level of significance (*p<0.05, **p<0.01).

**Table 3:** Mean value and significance of soil temperature, moisture and soil respiration from 2007 to 2009 between precipitation treatments in the pine forest (PF), the mixed forest (MF) and the broadleaf forest (BF), respectively.

Variable	Broadleaf forest (BF)		Mixed forest (MF)		Pine forest (PF)	
	DP	AP	DP	AP	DP	AP
Soil temperature	20.25^a^	20.39^a^	20.12^a^	20.29^a^	22.32^a^	22.52^a^
(°C)	±0.48	±0.48	±0.52	±0.51	±0.42	±0.41
Soil moisture	30.36^a^	27.40^b^	30.98^a^	28.46^b^	21.11^a^	18.13^b^
(% Vol.)	±1.21	±1.20	±1.10	±1.14	±0.97	±0.94
Soil respiration	3.08^a^	3.06^a^	3.25^a^	3.04^a^	2.54^a^	2.20^b^
(µmol CO_2_ m^−2^ s^−1^)	±0.11	±0.13	±0.12	±0.13	±0.11	±0.11

Table shows means and standard errors of soil temperature at 5 cm depth, soil moisture of the top 5 cm soil layer, and soil respiration rate under ambient precipitation (AP) and double precipitation (DP) treatments from the broadleaf forest, the mixed forest and the pine forest.

Mean values in each forest within a row with different letter have significant differences at α = 0.05 level.

Soil moisture was significantly influenced by precipitation treatments and varied among forest ecosystem types and years (Table 2). Soil moisture in both the DP and AP treatments showed strong variations in all three forests (Figure 2b). Soil moisture was maintained at about 29% vol. in the BF and the MF, but only 20% vol. in the PF over the observation period (Table 2; Figure 2b). The DP treatment slightly increased annual mean soil moisture by approximately 11.4% compared to the AP treatment.

### Effects of Precipitation Treatments on Soil Respiration, Soil Microbial Biomass and Fine Root Biomass

The soil respiration rate was significantly influenced by forest ecosystems and precipitation treatments, and the effects of precipitation treatments varied among the three forest ecosystems (Table 2). Soil respiration was significantly lower in the PF (2.37 µmol CO_2_ m^−2^ s^−1^), compared to that in the BF (3.07 µmol CO_2_ m^−2^ s^−1^) and MF (3.15 µmol CO_2_ m^−2^ s^−1^), averaged over three years of the experiment. The DP treatment increased mean annual soil respiration in the PF (15.4%), and did not show significant change in the BF or the MF.

**Figure 3:**
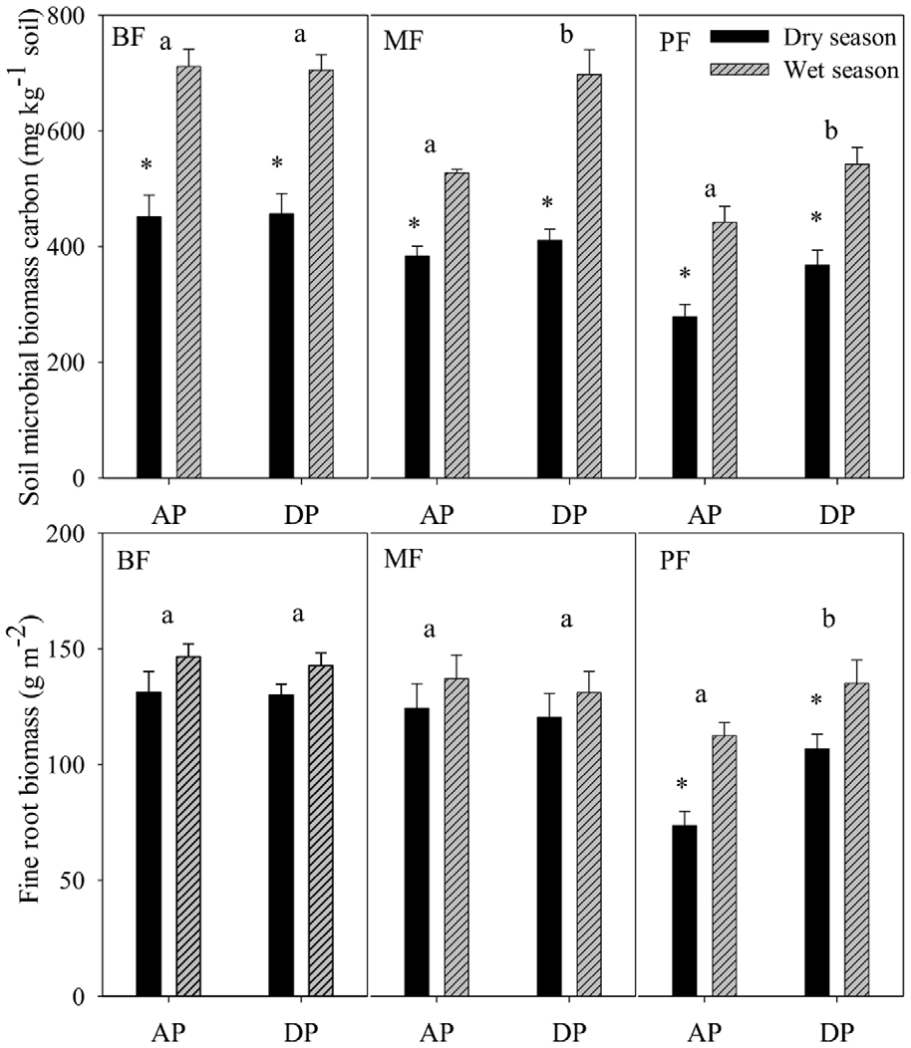
Soil microbial biomass carbon content and fine root biomass (diameter≤3 mm) under ambient precipitation (AP) and double precipitation (DP) treatments in the broadleaf forest (BF), the mixed forest (MF) and the pine forest (PF). Error bars are standard errors, sample size n = 6 for soil microbial biomass carbon content, sample size n = 3 for fine root biomass. Different letters in each forest denote significant difference (p<0.05) among precipitation treatments. *indicates significant difference between wet and dry seasons.

The responses of soil microbial biomass and fine root biomass to precipitation treatment also varied among forest ecosystems (Figure 3). Soil microbial biomass in the DP treatment increased by 19.0% and 24.0% in the MF and the PF, respectively, compared to the AP treatment (Figure 3), but did not change in the BF. The DP treatment enhanced soil microbial biomass in both the wet and dry seasons in the PF, but only in the wet season in the MF. The DP treatment increased fine root biomass by 31.2% in the PF, but not in the MF and the BF (Figure 3). Fine root biomass in the PF was enhanced in the dry season by the DP treatment.

### Effects of Precipitation Treatments on the Functional Relationships of Soil Respiration with Soil Temperature and Moisture

**Figure 4:**
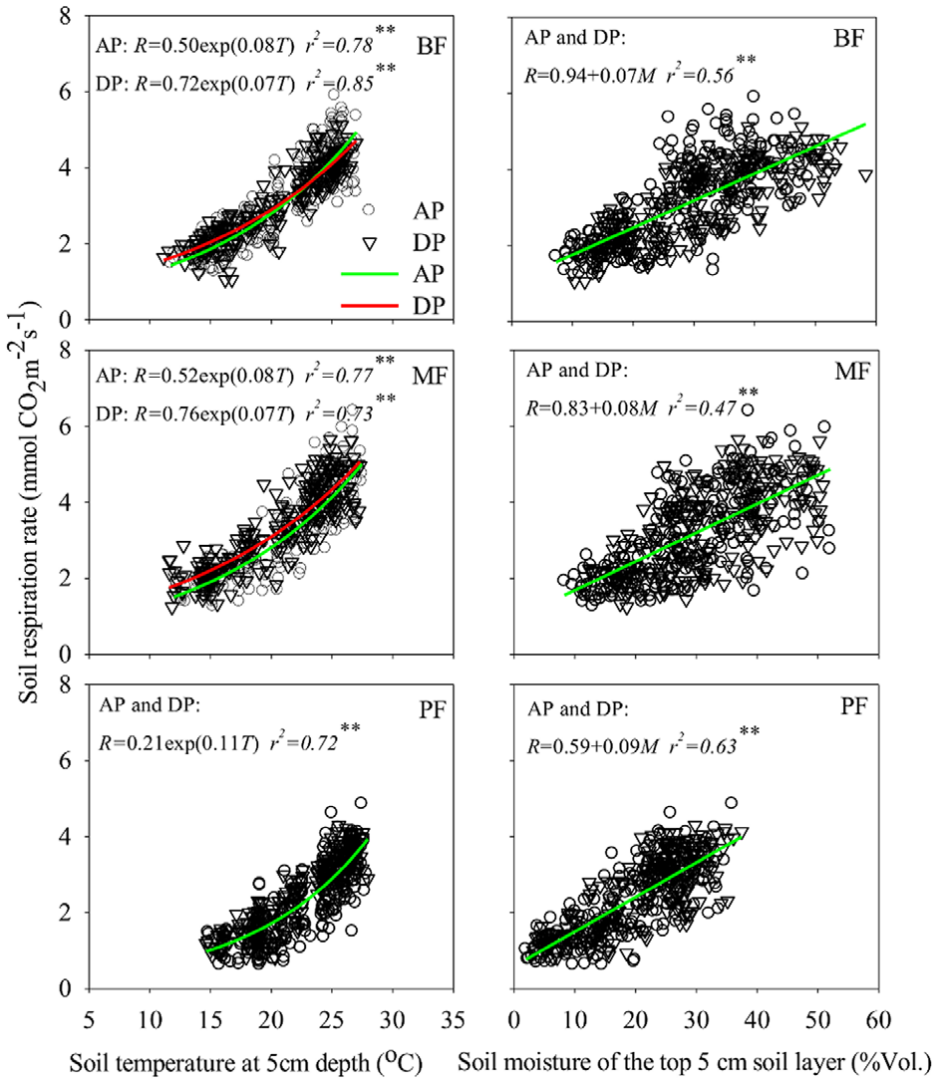
Relationship of soil respiration with soil temperature or soil moisture under ambient precipitation (AP) and double precipitation (DP) treatments in the broadleaf forest (BF), the mixed forest (MF) and the pine forest (PF). If fitted models aren’t significantly different between in the AP and DP treatments, one single model is fitted for all data. **indicates significant relationship at α = 0.01 levels.

Under both precipitation treatments and in all three forests, soil respiration responded exponentially to soil temperature and linearly to soil moisture (Figure 4). The DP treatment reduced soil temperature sensitivity in the BF and the MF, but not in the PF. Soil moisture sensitivity was not influenced by the DP treatment. Since soil temperature and soil moisture interactively regulate soil respiration, we considered both soil temperature and soil moisture and fit a combination model [30]. The best regression models explained 75–93% of soil respiration variations under two precipitation treatments in three forests (Table 4). The DP treatment decreased soil temperature sensitivities in the BF and the MF, but did not change soil moisture sensitivity. Basal soil respiration was enhanced under the DP treatment in both the BF and the MF. Under high temperature and heavy precipitation conditions, soil respiration under the DP treatment was lower than that under the AP treatment (Table 4), but in the PF, the DP treatment did not change the functional relationship of soil respiration with soil temperature and moisture developed under the AP control.

**Table 4:** Functional relationship and significant test of model parameters.

Forest	Treatment	*a*	*C*	*b*	*Q*_10_	*R* ^2^
Broadleaf forest	DP	0.7158±0.0267^a^	0.0088±0.0009^a^	0.0544±0.0020^a^	1.72	0.91^**^
	AP	0.5486±0.0358^b^	0.0082±0.0013^a^	0.0646±0.0033^b^	1.91	0.83^**^
Mixed forest	DP	0.8077±0.0458^a^	0.0068±0.0015^a^	0.0556±0.0029^a^	1.74	0.79^**^
	AP	0.5698±0.0395^b^	0.0064±0.0013^a^	0.0660±0.0035^b^	1.93	0.80^**^
Pine forest	DP	0.2541±0.0145^a^	0.0074±0.0008^a^	0.0786±0.0026^a^	2.19	0.93^**^
	AP	0.2691±0.0333^b^	0.0111±0.0026^a^	0.0657±0.0064^a^	1.93	0.75^**^

Relationship of soil respiration rate (*R*, µmol CO_2_ m^−2^ s^−1^) with soil temperature at 5 cm below the soil surface (*T*,°C) and soil moisture of the top 5 cm soil layer (*M*, % vol.) is developed using *R = *(*a+cM*)exp(*bT*) (parameter estimate ± standard error). *R* ^2^ in the table is the determination of coefficient, *Q_10_* = exp(10*b*) is temperature sensitivity coefficient, and slope *c* is soil moisture sensitivity. The treatments are: AP = ambient precipitation, DP = double precipitation. Different letters in each forest within a column denote significant difference (*p*<0.05) between the two precipitation treatments. **p<0.01. Numbers in bold indicates significant differences with the AP treatment.

## Discussion

The findings from our three-year precipitation manipulation experiment provide insights into the effects of precipitation increase on forest ecosystem soil respiration in subtropical monsoon areas and may have significant implications in modeling soil respiration. First, we found that unlike in arid and semi-arid ecosystems, soil respiration in the subtropical forests showed little response to precipitation increase, even when the precipitation was doubled. Second, we proposed to differentiate two reasons of soil respiration changes in response to precipitation increase (i.e., changes due to climate factor change and/or functional change) and demonstrated that different mechanisms may lead to different responses of soil respiration to precipitation treatments in different forest sites. The DP treatment increased soil moisture, enhanced basal soil respiration, but decreased soil temperature sensitivity in the BF and MF, resulting in no change in soil respiration. The increase in soil respiration in the PF under the DP treatment was solely caused by an increase in soil moisture, as no functional change was detected. Third, the slight increase in soil respiration under the DP treatment in the PF was supported by increases in soil microbial biomass and fine root biomass. As no changes in soil microbial biomass and fine root biomass were observed in the BF treatment and only slight change in soil microbial biomass in the MF, little change in soil respiration was observed in the MF and the BF. Our findings indicate that total soil respiration might not change much in the subtropical forests if precipitation increases in the future.

### Responses of Soil Respiration to Precipitation Treatments

Previous studies have indicated that the water status of an ecosystem may influence the direction of soil respiration to either reduction or increase in precipitation treatments [25]. In this study, we found 15.4% annual increase in soil respiration in the PF and no change of soil respiration in the BF and the MF (Table 3). Different responses might be attributed to differences in soil condition and vegetation at these study sites. Soil in the PF contains more sand, less clay, and more gravel, and had lower ambient soil moisture content than those in the BF and the MF (Table 1). Trees in the PF were younger and smaller in biomass and LAI [29]. As a result, we found that soil respiration in the PF was low, but showed a significant influence by precipitation increase. Responses of soil respiration to precipitation increase also varied among different studies. For example, the DP treatment resulted in an increase of 9.0% in soil respiration in a tallgrass prairie [10]. But a large increase of 31% in soil respiration was reported in arid and semiarid grassland with 30% increase in annual precipitation [36]. Results from a recent study indicated that soil respiration may be decreased under precipitation increase in a humid tropical forest [13].

### Functional Changes of Soil Respiration to Precipitation Treatments

Functional change of soil respiration to soil temperature/moisture under climate change is common and contributes to the responses of soil respiration in different ecosystems. A study in grasslands found that soil respiration was more sensitive to soil moisture than to soil temperature during prolonged drying cycles [52]. Ecosystems in xeric regions often possess lower soil respiration and higher soil moisture sensitivity than those in mesic regions [30], [53]. But the response of soil respiration to soil moisture change may be different in wet subtropical forests. We found that the DP treatment did not change soil moisture sensitivity, but decreased soil temperature sensitivity significantly in the BF and the MF ecosystems (Table 4). Many other studies have also found that soil respiration is insensitive to soil moisture unless that soil moisture is below levels at which metabolic activity decreases [20], [26], [54].

The lower temperature sensitivities under the DP treatment here may be due to the following two reasons. 1) Enhanced soil moisture under the DP treatment might decrease soil aeration and soil oxygen concentration [14], thus, more activation energy was needed to stimulate enzymatic rates [20]. Due to the subtropical monsoon climate, forests in the study site receive abundant heat, light, and water [55], [56]. Therefore, soils in these wet forests are often limited by soil oxygen concentration and nutrients, especially during the hot-humid season (April-September) [47]. 2) Greater leaching of dissolved organic carbon and nutrients under the DP treatment may reduce substrate availability [15], [57], and result in a decline in the Q_10_ values of soil respiration [8]. Previous work in this experiment has also shown that the active organic carbon, in particular particulate and light fraction organic carbon, often infiltrated to deeper soil layers with precipitation increase in the MF and BF [58], [59]. In the PF where soil was relative drier, the DP treatment stimulated fine root biomass and microbial activity (Figure 3). The greater soil microbial activity could release more nutrients from soil organic matter for fine root uptake, and increase soil respiration. The DP treatment in the BF and the MF did not stimulate soil microbe or fine root biomass, and caused little change in soil respiration in these forests.

### Environmental Factor Changes Alone may Contribute to Soil Respiration Changes Under Precipitation Treatments

Environmental factor changes induced by climate change alone could have significant influences on ecosystem responses. In this study, we found that the functional response of soil respiration to soil temperature and moisture in the PF under the DP treatment was not changed compared to the AP treatment (Table 4). However, increases in soil moisture under the DP treatment slightly enhanced soil respiration. A similar result was reported recently in a Mediterranean evergreen forest [37]. They found that when 27% of throughfall was excluded over three years, soil moisture was reduced by 7–10%. While the three-year throughfall exclusion did not change functional properties of the response of soil respiration to soil water content and soil temperature, soil respiration decreased by 11% due to the environmental factor change.

### Limitation of the Study

In this study, we selected three typical forest ecosystems in the south of China and tested the effects of precipitation increase on soil respiration. One shortcoming of the experimental design was unreplicated forest ecosystem types. While three replicated plots were employed for each precipitation treatment (i.e. DP and AP) at each forest ecosystem site, the forest types were not replicated. Thus, the inferences regarding the response differences among forest ecosystems should be read with caution. Further studies are needed to draw rigorous conclusions regarding forest ecosystem responses using replicated forest types.

### Conclusions

Using a three-year field experiment in subtropical forests in China, we demonstrated that soil respiration under the DP treatment was not changed in the BF and the MF, but slightly increased in the PF. The lower response of soil respiration was consistent with small or no change of fine root biomass and microbial biomass under the DP treatment. The different responses in the three forests were associated with both functional change and environmental factor change induced by the precipitation treatments. Changes in soil temperature sensitivity and basal soil respiration together with change in soil moisture help us understand soil respiration responses at different forest sites. The shift of soil temperature sensitivity and basal soil respiration under different precipitation regimes may have potentially significant implications for terrestrial ecosystem carbon cycling, and should be considered in terrestrial ecosystem models. Whether soil moisture sensitivity of soil respiration is changed by precipitation treatments, particularly drought, may warrant further study.
