# Correction: Effects of Precipitation Increase on Soil Respiration: A Three-Year Field Experiment in Subtropical Forests in China

**DOI:** 10.1371/annotation/1f49fc5e-e3f9-4b90-b555-97a54990ac3f

**Published:** 2012-10-15

**Authors:** Qi Deng, Dafeng Hui, Deqiang Zhang, Guoyi Zhou, Juxiu Liu, Shizhong Liu, Guowei Chu, Jiong Li

The authors Qi Deng and Deqiang Zhang should be noted as contributing equally to this work. 

